# Outcome of different facial nerve reconstruction techniques^☆^

**DOI:** 10.1016/j.bjorl.2015.12.010

**Published:** 2016-03-31

**Authors:** Aboshanif Mohamed, Eigo Omi, Kohei Honda, Shinsuke Suzuki, Kazuo Ishikawa

**Affiliations:** Akita Graduate School of Medicine, Head and Neck Surgery, Department of Otorhinolaryngology, Akita, Japan

**Keywords:** Facial nerve, Hypoglossal nerve, Nerve reconstruction, End-to-side anastomosis, Acoustic neuroma, Nervo facial, Nervo hipoglosso, Reconstrução de nervos, Anastomose término-lateral, Neuroma acústico

## Abstract

**Introduction:**

There is no technique of facial nerve reconstruction that guarantees facial function recovery up to grade III.

**Objective:**

To evaluate the efficacy and safety of different facial nerve reconstruction techniques.

**Methods:**

Facial nerve reconstruction was performed in 22 patients (facial nerve interpositional graft in 11 patients and hypoglossal-facial nerve transfer in another 11 patients). All patients had facial function House-Brackmann (HB) grade VI, either caused by trauma or after resection of a tumor. All patients were submitted to a primary nerve reconstruction except 7 patients, where late reconstruction was performed two weeks to four months after the initial surgery. The follow-up period was at least two years.

**Results:**

For facial nerve interpositional graft technique, we achieved facial function HB grade III in eight patients and grade IV in three patients. Synkinesis was found in eight patients, and facial contracture with synkinesis was found in two patients. In regards to hypoglossal-facial nerve transfer using different modifications, we achieved facial function HB grade III in nine patients and grade IV in two patients. Facial contracture, synkinesis and tongue atrophy were found in three patients, and synkinesis was found in five patients. However, those who had primary direct facial-hypoglossal end-to-side anastomosis showed the best result without any neurological deficit.

**Conclusion:**

Among various reanimation techniques, when indicated, direct end-to-side facial-hypoglossal anastomosis through epineural suturing is the most effective technique with excellent outcomes for facial reanimation and preservation of tongue movement, particularly when performed as a primary technique.

## Resumo

**Introdução:**

Não existe uma técnica de reconstrução do nervo facial que garanta a recuperação da função facial até o grau III.

**Objetivo:**

Avaliar a eficácia e segurança de diferentes técnicas de reconstrução do nervo facial.

**Método:**

Ao todo, 22 pacientes foram submetidos a reconstrução do nervo facial (enxerto com interposição do nervo facial em 11 pacientes e com transferência do nervo hipoglosso facial em 11 pacientes). Todos os pacientes apresentavam função facial de grau VI (de acordo com a classificação de House-Brackmann – HB) causada por trauma ou pela ressecção de um tumor. A reconstrução do nervo principal foi efetuada, exceto em sete pacientes, nos quais a reconstrução foi realizada entre duas semanas a quatro meses após a cirurgia inicial. O período de acompanhamento foi de, no mínimo, dois anos.

**Resultados:**

Para a técnica de enxerto com interposição de nervo facial, o grau de função facial obtido foi HB III em oito pacientes e HB IV em três pacientes. Sincinesia foi observada em oito pacientes e contratura facial com sincinesia em dois pacientes. Em relação à transferência do nervo hipoglosso facial com o uso de diferentes modificações, obtivemos função facial HB grau III em nove pacientes e HB grau IV em dois pacientes. Contratura facial, sincinesia e atrofia lingual foram observadas em três pacientes e sincinesia observada em cinco pacientes. No entanto, aqueles submetidos a anastomose primária direta hipoglosso-facial término-lateral apresentaram o melhor resultado, sem qualquer déficit neurológico.

**Conclusão:**

Entre as várias técnicas de reanimação, sempre que possível, a anastomose direta término-lateral hopoglosso-facial por meio de sutura epineural é a técnica mais eficaz, com excelentes resultados para reanimação facial e preservação do movimento da língua, especialmente quando realizada como técnica primária.

## Introduction

There are different surgical techniques for facial nerve reconstruction. Ideal repair consists of direct nerve repair, but sometimes a cable nerve graft is needed if a tension-free anastomosis cannot be achieved without a nerve graft. If this is not feasible, other techniques should be used, such as cross-facial nerve grafting, nerve muscle transposition, and cross-over motor cranial nerve substitution.^1^ In 1903, Körte described the anastomosis of the facial nerve (VII nerve) to the side of the hypoglossal nerve (XII nerve).^2^ In 1979, Conley et al. described the first end-to-end VII–XII suture. Several modifications have since been reported, including “split” XII–VII transfer, in which 30% of the hypoglossal nerve is divided and secured to the lower division of the facial nerve.^3^ In 2000, May et al. described the VII–XII jump graft. This involves end-to-side neurorrhaphy using a donor cable graft.^4^ In 1997, Atlas and Lowinger described a new modification in which the facial nerve was mobilized from the second genu and reflected inferiorly for direct anastomosis to the hypoglossal nerve.^5^ We present our results of facial function in a group of patients who developed facial paralysis due to different causes, along with their long term outcomes, using different techniques of nerve reconstruction, including the latest end-side facial hypoglossal nerve anastomosis.

## Methods

**Figure 1:**
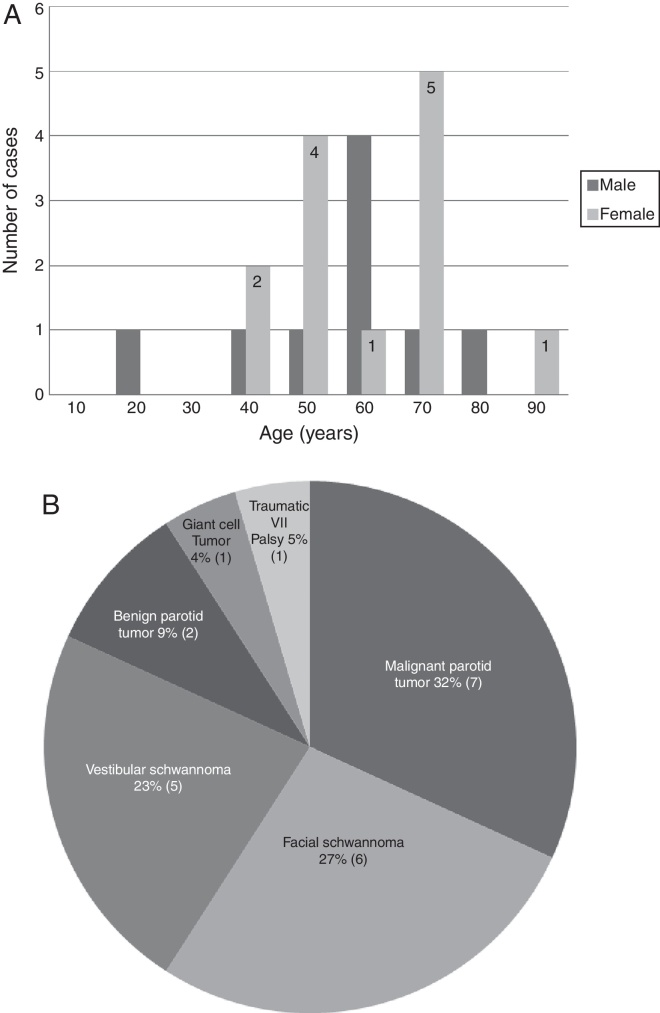
Age distribution and causes of facial paralysis. (A) A diagram indicating the number of cases in different age groups according to the gender, the dark columns refer to males and the light numbered columns refer to females. (B) A graph for a variety of diseases treated for facial paralysis.

We reviewed the medical records of 22 patients operated for facial nerve paralysis at our institution between 1991 and 2013. The average age was 53.5 years (18–81 years) (Fig. 1A). Facial paralysis was due to different reasons (Fig. 1B). The clinical assessment of facial function was grade VI in all patients according to House-Brackmann grading system, because the facial nerve was either severed intra-operatively by trauma or facial paralysis developed in spite of the maintenance of the integrity of the facial nerve. All data regarding age and sex of the patients, and etiology or duration of the paralysis and long term results (at least two years follow-up) were obtained (Table 1, Table 2). All patients were evaluated regarding the facial nerve function, facial contracture, synkinesis and tongue atrophy. All patients signed an informed written consent.

**Table 1:** Patients’ characteristics and results in facial nerve interpositional graft technique.

	Age (years)	Sex	Pathology	Interval	HB score (follow-up months)	Complications
1	49	F	Acoustic tumor	–	III (100)	Facial contracture, synkinesis
2	74	M	Traumatic VII palsy	1 month	III (44)	Synkinesis
3	18	M	Facial Schwannoma	–	IV (36)	Synkinesis
4	81	F	Benign parotid tumor	–	IV (43)	Facial contracture, synkinesis
5	48	F		–	III (72)	Synkinesis
6	60	M	Malignant parotid tumor	–	III (48)	Synkinesis
7	63	F		–	III (46)	
8	66	F		–	III (60)	
9	67	M		–	III (54)	
10	32	F		–	III (84)	–
11	49	F		–	IV (62)	Synkinesis

**Table 2:** Patients’ characteristics and results in facial-hypoglossal nerve transfer technique.

	Age (years)	Sex	Pathology	Technique	Interval	HB Score (follow-up months)	Complications
1	47	F	Acoustic tumor	End-end	4 months	III (114)	Facial contracture, synkinesis, tongue atrophy
2	69	F	VII Schwannoma		–	III (60)	
3	41	M			2 weeks	III (132)	
4	31	F	VII Schwannoma	Direct end-side (epineural suturing)	–	III (64)	±Synkinesis
5	37	M				III (63)	–
6	57	M	Acoustic tumor		3 weeks	III (37)	
7	69	F			1 month	III (71)	
8	51	M	Malignant parotid tumor	End-side interposition graft (VII peripheral branches XII)	–	III (80)	±Synkinesis
9	51	M	VII Schwannoma	End-side interposition graft (VII main trunk XII)		IV (54)	
10	61	F	Giant cell tumor	Side-side (epineural suturing)	3 weeks	IV (24)	Synkinesis
11	58	F	Acoustic tumor		2.5 months	III (45)	±Synkinesis

### Operative techniques

**Figure 2:**
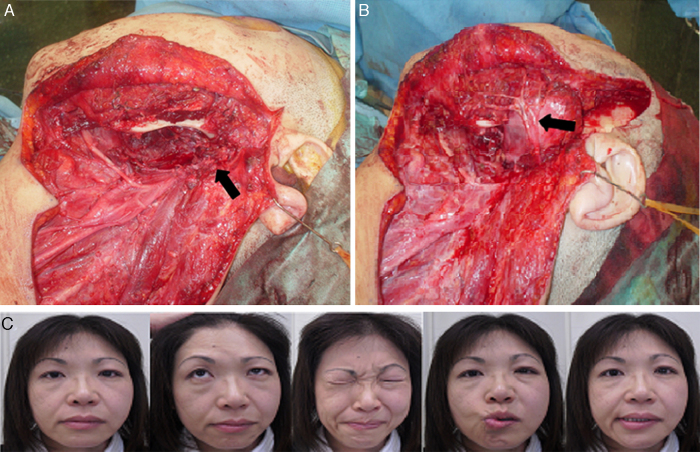
Intraoperative view and result of facial nerve interpositional graft technique (Patient 10). (A) The patient had left extended total parotidectomy and left radical neck dissection, the black arrow points to the facial nerve stump. (B) Primary facial nerve reconstruction with interposition of a nerve graft using cutaneous cervical nerves (marked by black arrow) was done. (C) Improvement in facial function up to HB grade III with no synkinesis.

We have used different techniques for facial nerve reconstruction. In eleven cases, we have performed facial nerve interpositional graft where a cable graft (great auricular nerve or cervical cutaneous nerves) was employed to span the distance between the proximal and distal segments of the facial nerve as in the patient number 10 (Fig. 2A and B).

In the other eleven cases where the previous method was not feasible, we have used hypoglossal-facial nerve transfer (XII–VII Cross-over) techniques; the classic XII–VII procedure is performed via a modified Blair parotidectomy incision. The main trunk of the facial nerve and the pes anserinus are identified using standard facial nerve landmarks, such as the tragal pointer. The hypoglossal nerve is then located in its ascending portion, deep to the posterior belly of the digastrics muscle, sharply transected and reflected superiorly to meet the facial nerve. The facial nerve is transected at the stylomastoid foramen, and the entire distal trunk is reflected inferiorly and secured to the hypoglossal nerve with 5–7 stitches using 9–0 prolene suture (Ethicon, Johnson & Johnson Medical; Norderstedt, Germany).

In the direct VII–XII end-to-side anastomosis technique, once the exposure has been obtained, the mastoid portion of facial nerve is mobilized, sectioned at the second genu, and rotated inferiorly into the neck after removal of the mastoid tip. Then, the connective tissue of the hypoglossal nerve and facial nerve stump is removed to expose the epinerium. The epineurium of the hypoglossal nerve is then incised and sutured to the epineurium of the facial nerve directly with 5–7 stitches using 9–0 prolene sutures. Two patients had primary reconstruction and secondary reconstruction was performed in other two.

The jump graft modification technique was used as a primary reconstruction in two cases, in which the facial nerve is cut at the stylomastoid foramen. Then, the great auricular nerve is harvested and used as a cable graft to span the distance between the main trunk of facial nerve and the hypoglossal nerve. When the main trunk was invaded by tumor as in the patient number 8, we used the cutaneous cervical nerves to span the VII peripheral branches and the side of hypoglossal nerve.

**Figure 3:**
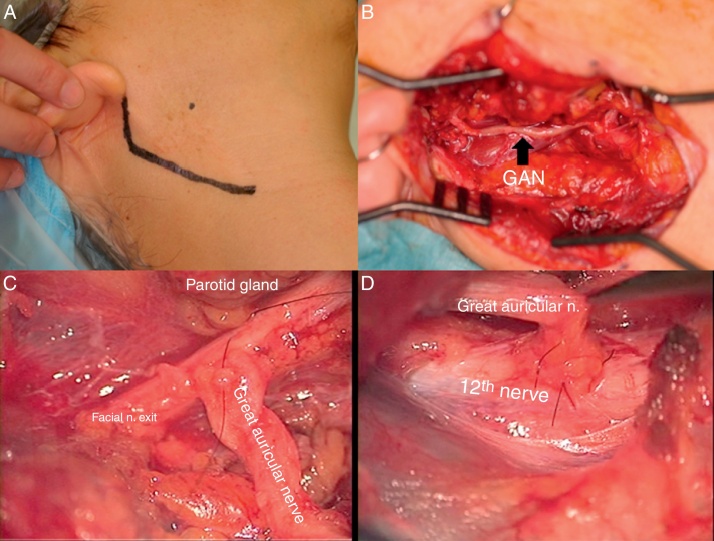
Intraoperative view of side-to-side FHA technique (Patient 11). (A) The modified Blair's incision is marked in black line. (B) Interposition of great auricular nerve (GAN) between main trunk of facial nerve and hypoglossal nerve. (C) Microscopic view of epineural suturing of GAN to the side of facial nerve trunk. (D) Microscopic view of epineural suturing of GAN to the side of hypoglossal nerve.

As the last modification, we made an epineural window through the sheath of both facial and hypoglossal nerves. Then, the great auricular nerve was harvested, interpositioned between both sides of facial and hypoglossal nerves and sutured to both nerves using an epineural suturing technique (Fig. 3). In both cases, the surgical procedures were a secondary reconstruction.

## Results

Considering that the shorter the interval between paralysis and reconstruction, the better the outcomes will be, in 15 patients we performed a primary facial nerve reconstruction just after the resection of the original pathology (vestibular schwannomas, facial schwannomas, parotid tumor, giant cell tumor and parapharyngeal tumor). In the remaining seven cases, the facial nerve reconstruction was performed as a secondary procedure after the stabilization of the patient's general condition; the waiting period ranging from a two-week to a four-month interval after the facial paralysis. Of the 22 patients treated, 11 patients had facial nerve interpositional graft, three patients had end-to-end VII–XII anastomosis, four patients had direct end-to-side VII–XII anastomosis, two patients had end-to-side VII–XII interpositional graft, and two patients had side-to-side VII–XII anastomosis.

After two years of follow-up, eight (73%) of the facial nerve interpositional graft cases improved their facial function up to HB-grade III, and three cases (27%), up to HB-grade IV. Also, eight (73%) of them had synkinesis, two cases (18%) had facial contracture and synkinesis, and one case (9%) had no problem (Fig. 2C).

We performed an end-to-end VII–XII anastomosis in three cases. The facial function was improved up to grade III in all cases. Unfortunately, all of them developed facial contracture, synkinesis and tongue atrophy.

**Figure 4:**
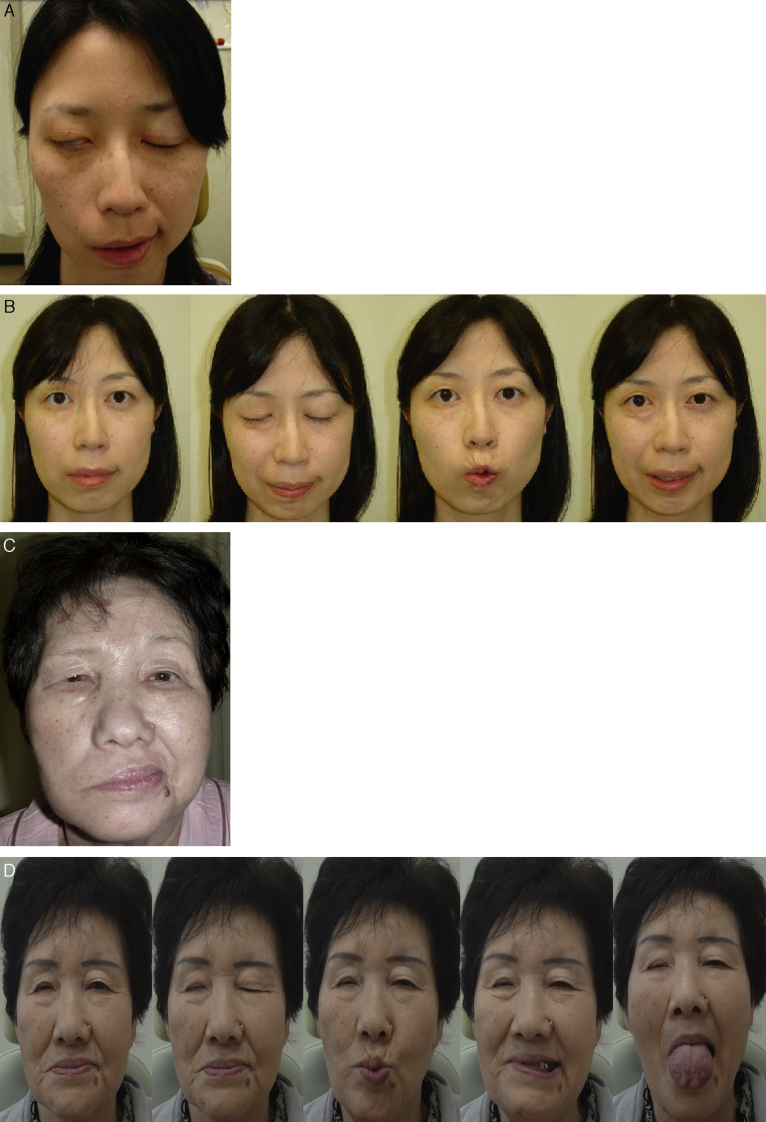
Pre and post-operative result of direct end-to-side FHA technique (Patient 4 and 7) (Patient 4; Facial N Res Jpn 30: 186–189, 2010). (A) Preoperative photo (Patient 4); (B) postoperative facial function shows HB grade III with mild synkinesis (Patient 4); (C) preoperative photo (Patient 7); (D) postoperative facial function shows HB grade III with no synkinesis and normal tongue movement (Patient 7).

Direct end-to-side VII–XII anastomosis was performed in four cases; all four achieved improvement in the facial function up to HB-grade III. Remarkably, three of them (75%) did not show any complications (Fig. 4), while one case showed mild synkinesis. On the other hand, primary end-to-side VII–XII jump graft was performed in two patients and both achieved improvement in their facial function up to grade III but, synkinesis developed in both cases.

**Figure 5:**
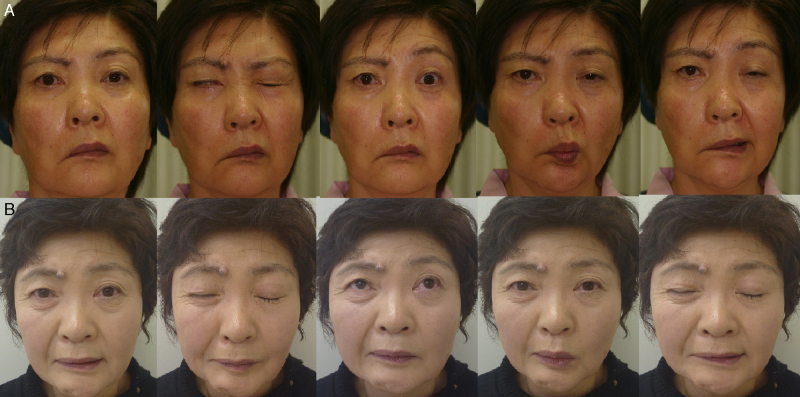
A case of right acoustic neuroma (Patient 11). (A) Preoperative facial function shows HB grade VI. (B) Postoperative facial function shows HB grade III with mild synkinesis.

Side-to-side VII–XII anastomosis was performed in two cases; the facial function improved up to HB-grade III in one case and up to grade IV in the other patient. Also, synkinesis developed in both cases (Fig. 5).

## Discussion

Although multiple techniques have been proposed to repair the loss of facial nerve function, each with its own indications, contraindications and complications, there is no method that guarantees facial function recovery up to House-Brackmann grade III.^6^

When the proximal stump of the facial nerve is accessible, the injury is best treated with an end-to-end anastomosis or with the interposition of a nerve graft (if a gap of more than 1 cm is observed) during the surgical intervention itself, since maximal nerve reanimation is achieved when the central portion is accessible and is connected to the distal branch of the same nerve.^5^

In certain cases, the proximal part of the nerve may have to be resected close to the brainstem, where nerve repair is impossible by means of an end-to-end suture. In these cases, Facial-Hypoglossal Anastomosis (FHA) is a technique that is frequently used to provide proper nerve impulse to the distal remnant of the facial nerve.^7^ This technique is also indicated when, despite anatomical preservation of the facial nerve, there is complete facial paralysis and there is no functional recovery, as long as the muscles of the mimetic nerves are still functioning,^8^ as we found in patient number 11. Most authors agree that the best results are achieved by using the hypoglossal nerve due to its presence close to the extra-temporal facial nerve, its diameter and its dense population of myelinated motor axons, which prevent extensive tissue dissection and allow for a tensionless nerve suture. Furthermore, due to the neighboring brainstem nuclei of the hypoglossal and facial nerve and neuronal brain plasticity, favorable postoperative outcomes in terms of function have been described.^9^, ^10^, ^11^

The classic end-to-end FHA is an effective procedure with excellent tone at rest. However, transection of the hypoglossal nerve causes ipsilateral hemiglossal atrophy. On the other hand, the difference in axonal load between the hypoglossal nerve and the facial nerve causes synkinesis and spasm,^12^ as we found in our cases.

In order to solve this problem, another modification was used where we performed an end-to-side VII–XII interpositional graft. A great auricular nerve and cervical cutaneous nerves were used as a cable graft. But the presence of two neurorrhaphies may influence the reinnervation quality and time,^13^, ^14^ so we found synkinesis in both patients.

Given the fact that the length of the mastoid facial nerve is around 16.4 mm (range 15.2–18.6 mm), and if the distance from the stylomastoid foramen to the facial nerve bifurcation, measuring 18.9 mm (range 16–20.6 mm), is added to that figure, a total length of 35.3 mm is obtained. This is longer than the distance between the bifurcation of the facial nerve and the location at which the hypoglossal nerve turns toward the tongue, which measures 31.6 mm (range 27.6–35.8 mm), and should be sufficient for tensionless nerve anastomosis.^15^ Also, the presence of one neurorrhaphy makes it more easier for regenerating fibers to pass through one gate, so we have employed the direct end-to-side FHA in four cases where the proximal part of facial nerve was not accessible.

In some cases, where there is postoperative facial paralysis in spite of preservation of the anatomical integrity of facial nerve, side-to-side FHA technique was employed to enhance the recovery process. The best results of cranial nerve XII–VII anastomosis were obtained when the procedure was performed within two months after nerve damage, and a denervation time of 6–12 months guarantees at least satisfactory results, but, in cases of longer denervation time, the vitality of the affected musculature has to be examined thoroughly.^16^ So, we performed primary reconstruction in most cases and, as soon as possible, in the other cases.

## Conclusion

Cases can sometimes be encountered in which the facial nerve is unfortunately sacrificed, with the length of the remnant facial nerve varying between cases, and we are expected to select the best procedure among various options to obtain optimal facial reanimation. Direct end-to-side VII–XII anastomosis through epineural window was the best option. The merits of direct end-to-side anastomosis in terms of both procedure and outcomes should be kept in mind as a potentially useful method for facial reanimation.

## Conflicts of interest

The authors declare no conflicts of interest.
